# Adipocyte-Like Differentiation in a Posttreatment Embryonal Rhabdomyosarcoma

**DOI:** 10.1155/2015/406739

**Published:** 2015-12-10

**Authors:** Dana Balitzer, Timothy H. McCalmont, Andrew E. Horvai

**Affiliations:** ^1^Department of Pathology, University of California, San Francisco, 505 Parnassus, M580, San Francisco, CA 94143, USA; ^2^Departments of Pathology and Dermatology, University of California, San Francisco, 1701 Divisadero Street, Suite 280, San Francisco, CA 94115, USA; ^3^Department of Pathology, University of California, San Francisco, 1825 4th Street, M2354, San Francisco, CA 94143, USA

## Abstract

We describe a 16-year-old boy with rhabdomyosarcoma, consistent with embryonal subtype, of the lower extremity who received systemic neoadjuvant chemotherapy and subsequent excision. Microscopic sections of the postchemotherapy excision demonstrated diffuse, prominent, and immature adipocyte-like differentiation, in addition to skeletal muscle differentiation. Adipocyte-like differentiation was confirmed by a combination of positive Oil Red O and adipophilin immunohistochemical staining. To our knowledge, this represents the first report of an unusual phenomenon of differentiation of a soft tissue rhabdomyosarcoma into adipocyte-like cells after chemotherapy.

## 1. Introduction

Rhabdomyosarcoma is an aggressive mesenchymal neoplasm, most common in children, that frequently requires multimodality therapy consisting of surgery and chemotherapy [1]. So-called “cytodifferentiation” after neoadjuvant chemotherapy is a well-described phenomenon in rhabdomyosarcoma and consists of more prominent rhabdomyoblasts and decreased primitive, proliferating cells [2–4]. Rhabdomyosarcoma with adipocyte-like cells has been rarely observed in unusual ovarian carcinosarcomas [5]. However, to our knowledge, the present case represents the first example of a soft tissue rhabdomyosarcoma with adipocyte-like cells after chemotherapy.

## 2. Case Presentation

One year prior to presentation, a 16-year-old boy with no significant medical history noticed a lump, which progressively enlarged over the course of several months, at his posterior left knee. Magnetic resonance imaging demonstrated a 13-centimeter mass in the left posteroinferior thigh, centered in the region of the semitendinosus muscle and extending to the long head of the biceps femoris muscle.

**Figure 1:**
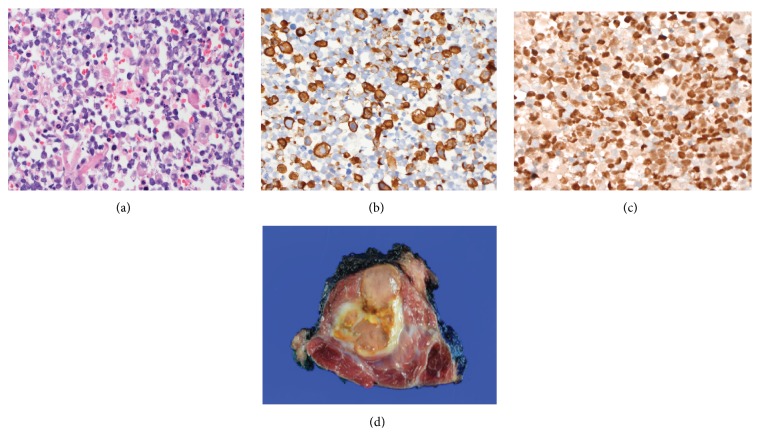
Gross and microscopic findings. (a) Hematoxylin and eosin (H&E) stain from fine-needle aspiration sample, prior to chemotherapy treatment, reveals a heterogeneous population of ovoid to slightly spindled cells (H&E, ×100 original magnification), (b) a subset of tumor cells are positive for desmin immunostain (×400 original magnification), and (c) most tumor cells are positive for myogenin (myogenin immunostain, ×400 original magnification). (d) Gross appearance of resected tumor after adjuvant chemotherapy treatment.

A fine-needle aspiration of the tumor demonstrated a heterogeneous population of ovoid to tapered spindled cells with variable amounts of densely eosinophilic cytoplasm and occasional multinucleated cells (Figure 1(a)). No lipogenic differentiation was noted on any of the smears or cell block. Immunohistochemical stains for desmin (Figure 1(b)) and myogenin (Figure 1(c)) were positive, whereas staining for CD68, keratin, S-100, SOX-10, HMB-45, and Melan-A was negative (not shown). A diagnosis of rhabdomyosarcoma was made. Fluorescence in situ hybridization was negative for a* FOXO1* 13q14 rearrangement. Based on the cytomorphology and lack of* FOXO1* rearrangement, an embryonal subtype was favored. He received chemotherapy per intermediate risk protocol ARST0531 with vincristine/irinotecan for 10 weeks.

**Figure 2:**
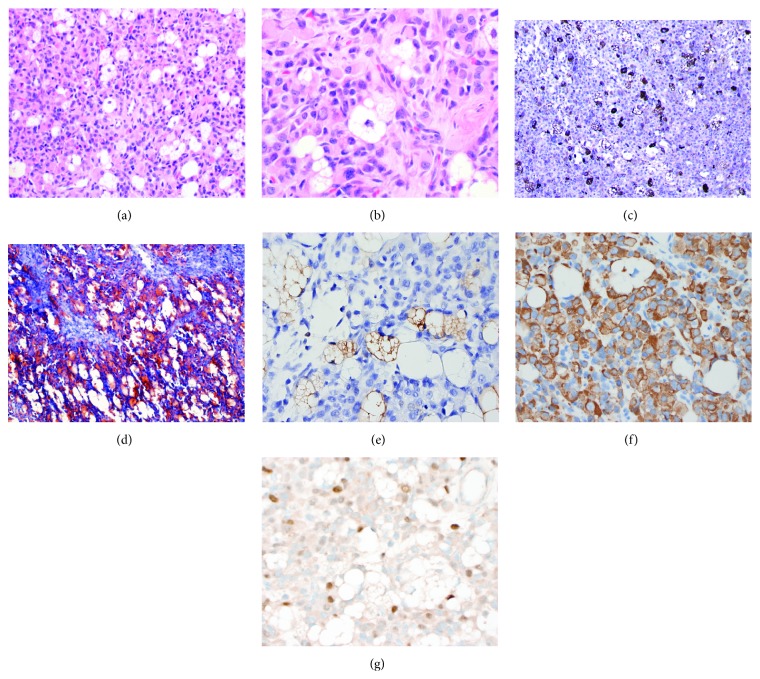
Histochemical and immunohistochemical studies in posttreatment rhabdomyosarcoma. (a) Microscopic appearance of resected tumor after adjuvant chemotherapy, demonstrating diffuse sheets of round tumor cells (H&E, ×200 original magnification) and (b) vacuolated adipocyte-like cells (H&E, ×400 original magnification). (c) Adipophilin demonstrates strong membrane staining within adipocyte-like cells (adipophilin immunostain, ×100 original magnification). (d) Oil Red O histochemical stain highlights intracytoplasmic lipid within the adipocyte-like cells (Oil Red O, ×200 original magnification). (e) The adipocyte-like cells stain with S-100 protein (S-100 protein immunostain, ×400 original magnification). (f) A subset of vacuolated adipcoyte-like cells show focal cytoplasmic desmin positivity (desmin immunostain, ×400 original magnification) but myogenin (g) is negative in vacuolated cells, staining only the nuclei of rhabdomyoblasts (myogenin immunostain, ×400 original magnification).

Following chemotherapy, the tumor was completely excised. Grossly, the mass was mixed, cystic and solid, and separated from the surrounding skeletal muscle by a thin white capsule (Figure 1(d)). Routine hematoxylin and eosin (H&E) sections demonstrated diffuse sheets of round tumor cells with variable amounts of dense, eosinophilic rhabdomyoblastic cytoplasm with large nuclei (Figure 2(a)). Diffusely throughout all sections of the tumor, a subset of cells showed a spectrum of vacuolation. The vacuolation ranged from small intracytoplasmic vacuoles to cells with multiple vacuoles indenting the nucleus thereby resembling adipocytes or lipoblasts (Figure 2(b)). The vacuolated cells were positive for adipophilin (Figure 2(c)), Oil Red O (Figure 2(d)), and S-100 protein (Figure 2(e)). Adipophilin is a ubiquitous component of lipid droplets and is a specific marker for lipid accumulation, which has been reported in cells of lactating mammary epithelium, adrenal cortex, male reproductive system (Sertoli and Leydig cells), steatotic hepatocytes in alcoholic cirrhosis, and liposarcomas [6]. The vacuoles in the adipocyte-like cells did not stain with periodic acid-Schiff, with or without diastase (not shown). An immunostain for desmin demonstrated staining within the cytoplasm of adipocyte-like cells (Figure 2(f)), while immunostaining for myogenin was negative within the nuclei of adipocyte-like cells (Figure 2(g)).  Table 1 lists the antibody sources and dilutions used. Cystic change, scattered histiocytes, and extracellular hemosiderin consistent with treatment effect were also present. Mitotic activity was low (1 mf/10 hpf). No coagulative necrosis was identified.

## 3. Discussion

Rhabdomyosarcoma is the most common soft tissue malignancy in children and adolescents and consists of a biologically and genetically diverse group of sarcoma [7]. Standard treatment regimens for rhabdomyosarcoma include local control with surgery or radiation therapy in conjunction with multiagent chemotherapy [1]. When the tumor is locally advanced, most protocols recommend a biopsy followed by neoadjuvant chemotherapy prior to excision [8].

Several studies have described the range of microscopic treatment related changes in rhabdomyosarcoma. Nonspecific treatment effect, such as necrosis, macrophage infiltrate, fibrosis, and skeletal muscle atrophy are commonly observed. A decrease in proliferative activity is also often present [9]. Rhabdomyosarcoma can also undergo histologic differentiation after treatment. Specifically, rhabdomyoblasts and strap cells become more prominent [2] while undifferentiated, primitive round cells become less conspicuous. Some authors have suggested that these apparently differentiated neoplastic cells may represent terminally differentiated elements withdrawn from the cell cycle after chemotherapy [10]. The presence of so-called postchemotherapy “cytodifferentiation” is thought to be more frequent in embryonal and botryoid rhabdomyosarcoma than other subtypes [3, 4].

In addition to the phenomenon of cytodifferentiation, vacuolated cells have been previously noted in cases of treated rhabdomyosarcoma. Coffin et al. described the presence of vacuolated cells in a rhabdomyosarcoma [9]. Likewise, a case report of an ovarian carcinosarcoma with pleomorphic rhabdomyosarcomatous differentiation noted the presence of “pseudolipoblasts” after neoadjuvant chemotherapy [5]. The presence of cells with morphology intermediate between rhabdomyoblasts and multivacuolated adipocyte-like cells suggested that the chemotherapy induced differentiation in the rhabdomyosarcomatous component of the carcinosarcoma, representative of a posttreatment phenomenon [5].

In our differential diagnosis, we also considered the possibility of clear cell rhabdomyosarcoma, or glycogen-rich rhabdomyosarcoma [11]. However, several lines of evidence argue against this possibility. First, clear cell rhabdomyosarcoma has not been reported as a treatment related change. Second, the histochemical and immunohistochemical features of the posttreatment excision support lipid accumulation rather than glycogen in the vacuolated cells. Rare examples of lipid-rich rhabdomyosarcomas have also been described, in untreated tumors but not as a morphologic change induced by chemotherapy [12, 13]. The presence of S-100 protein immunostaining, though not highly specific, is at least supportive of adipocytic differentiation. Furthermore, regenerating skeletal muscle can mimic rhabdomyosarcoma and sometimes demonstrate vacuolation. However, in the present case, the resection lacks other characteristic features of muscle regeneration such as sarcolemmal ghosts or necrotic myofibers [14]. We also considered the possibility that lipogenic differentiation was already present, but not sampled, in the FNA biopsy. Although it is impossible to completely exclude this possibility, the FNA yielded abundant cells using a “cone biopsy” technique that samples multiple regions of tumor [15, 16]. Yet, the biopsy contained no vacuolated cells whereas such cells were present diffusely in every section of the excision specimen. Finally, the decrease in density of myogenin positive cells after chemotherapy suggests a different expression pattern in the posttreated tumor compared to the pretreatment biopsy.

Rhabdomyoblastic differentiation is well described in a variety of mesenchymal tumors, including malignant mixed Mullerian tumors of the uterus and carcinosarcomas, as well as neuroectodermal neoplasms such as malignant Triton tumor or medulloblastomas. Intriguingly, rhabdomyoblastic differentiation has been reported in liposarcoma [17, 18], but the reverse (i.e., differentiation of rhabdomyoblasts into lipoblasts or adipocytes) appears extremely rare. Adipocyte-like cells have been previously reported in a primary ovarian rhabdomyosarcoma [19]. Recent cell culture studies suggest that rhabdomyosarcomas can arise from adipocyte progenitors via Smd activation, providing a model whereby embryonal rhabdomyosarcoma can arise in sites devoid of skeletal muscle [20]. Lineage tracing and clonal analysis studies have demonstrated that adult skeletal muscle stem cells are bipotential and can give rise to brown adipogenic and myogenic progenitors presumably under the correct genetic and epigenetic mechanisms [21].

To our knowledge, this is the first reported case of a soft tissue rhabdomyosarcoma with evidence to support adipocyte-like cells as a posttreatment phenomenon and expand the spectrum of cytodifferentiation that can be observed in treated rhabdomyosarcoma.

## Figures and Tables

**Table 1:** Antibody sources and dilutions.

Antibody	Source	Clone	Dilution
Adipophilin	Cell Marque, Rocklin, CA	Polyclonal	Undiluted
Desmin	Cell Marque	D33	1 : 5
Myogenin	Cell Marque	F5D	Undiluted
S-100 protein	DAKO, Carpinteria, CA	Polyclonal	1 : 2000
